# Miliary Tuberculosis in a Young Patient? Let's Not Forget the Lung Adenocarcinoma!

**DOI:** 10.7759/cureus.10058

**Published:** 2020-08-26

**Authors:** Zeeshan Zia, Qasim Z Iqbal, Naureen Narula, Saud Bin Abdul Sattar, Muhammad Rafay Khan Niazi

**Affiliations:** 1 Internal Medicine, Northwell Health-Staten Island University Hospital, New York, USA; 2 Internal Medicine, Allama Iqbal Medical College, Lahore, PAK; 3 Internal Medicine, King Edward Medical University/Mayo Hospital, Lahore, PAK

**Keywords:** lung adenocarcinoma, imaging, miliary tuberculosis

## Abstract

Lung cancer is one of the most common cancers diagnosed every year and accounts for a major percentage of cancer incidence and mortality annually, especially in men. Lung adenocarcinoma is a subtype of non-small cell lung cancer (NSCLC), which is the most common type of lung cancer found in smokers and nonsmokers alike. It is known to have diverse CT chest findings ranging from: ground-glass opacities (GGOs) with partially solid nodules, to single or multiple solid nodules that can be either central or peripheral, to thin-walled cystic lesions. Lesions are usually solitary but may be multifocal. Rarely, these lesions can be calcified or demonstrate cavitation, which can make it difficult to distinguish from an infectious disease like tuberculosis, pneumonia, or even fungal infections. Here, we present a case of a 36-year-old Asian male with no significant past medical history, except a 16-pack-year smoking history and recent deployment to Afghanistan, that initially presented with a chief complaint of cough of three-week duration. His CT scan showed innumerable bilateral pulmonary nodules within both lung fields with a miliary type appearance that ultimately turned out to be adenocarcinoma.

## Introduction

Lung cancer is the second most common cancer and accounts for 14% of new cancers every year. It is considered the most frequent cause of major cancer incidence and mortality in men. Non-small cell lung cancers (NSCLCs) account for around 85% of cases and of these adenocarcinomas are most common. The radiological appearance of peripheral lung adenocarcinomas encompasses a spectrum from ground-glass nodules (GGNs) to solid mass lesions on CT, reflecting their heterogeneous histological subtypes. This imaging can have a major impact on important outcomes such as patient mortality, and survival rates. Typically lung adenocarcinomas are peripherally located solitary lesions. Sometimes though the tumor might resemble an infectious pathology and hence could be seen on the imaging as a consolidation, a ground-glass opacity (GGO), or even both. Amongst the various types of lung cancers, these appearances are more commonly seen in patients with lung adenocarcinoma. We present an interesting clinical case of a rare atypical presentation of lung adenocarcinoma as seen in the imaging.

## Case presentation

**Figure 1:**
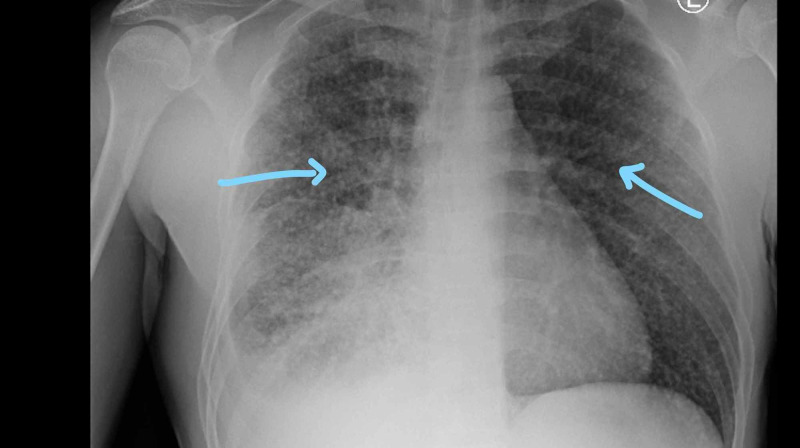
Chest x-ray showing diffuse bilateral opacities Arrows pointing towards diffuse bilateral opacities

**Figure 2:**
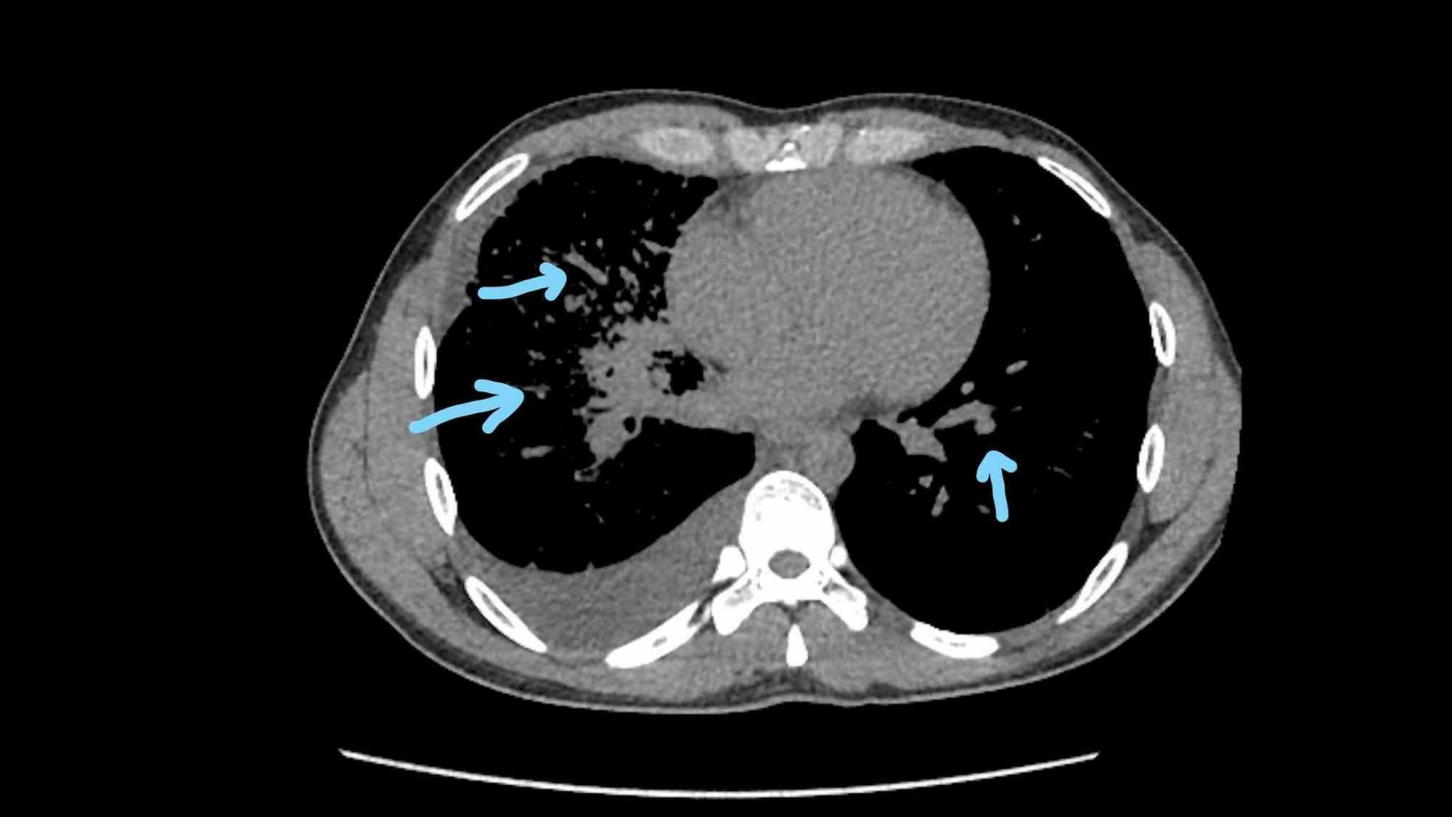
CT scan showing a diffuse bilateral miliary pattern Arrows pointing to bi-lateral multiple ground glass nodular opacities showing a miliary pattern

**Figure 3:**
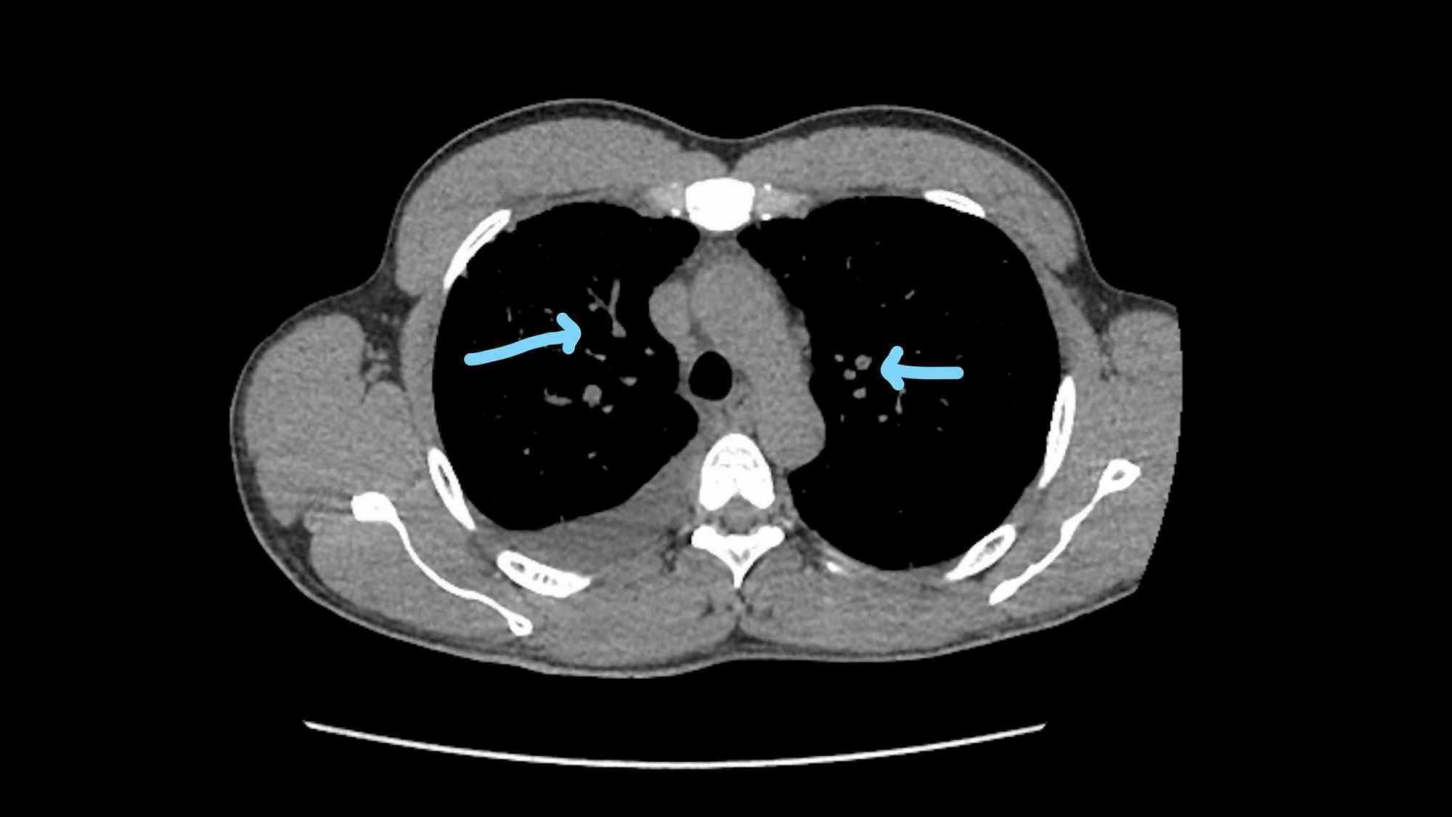
Bilateral tumor seeding Arrows pointing to diffuse miliary spread of lung adenocarcinoma

**Figure 4:**
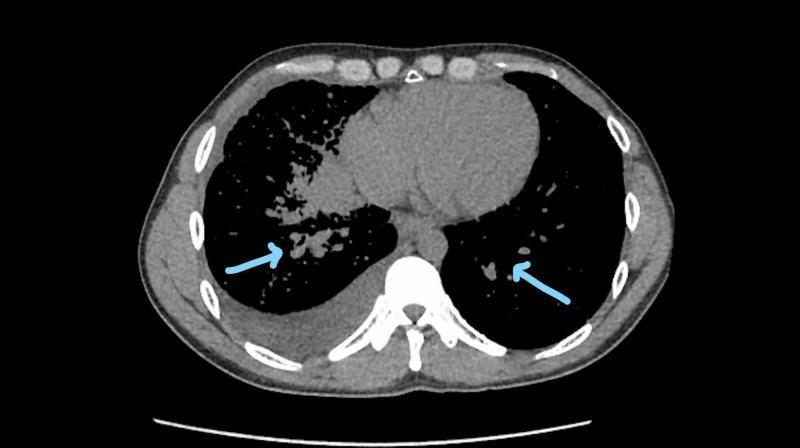
Bilateral pulmonary nodules Arrows point towards bilateral pulmonary nodules

**Figure 5:**
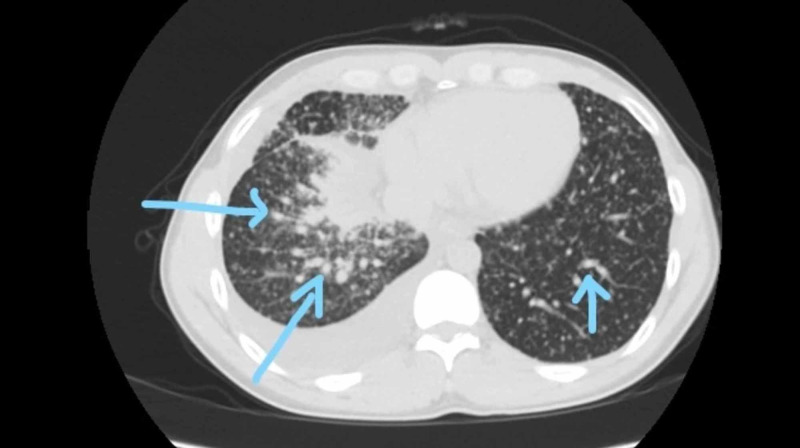
Lung window view of bilateral pulmonary nodules Arrows point towards a miliary pattern indicating bilateral pulmonary nodules

A 36-year-old Asian male with no past medical history presented to the ED with a chief complaint of non-productive cough of three-week duration. The patient reported a 16-pack-year smoking history and quit six months prior to presentation. On initial assessment, he denied any fever, shortness of breath, night sweats, weight loss, chest pain, or any significant weight loss. He reported his wife recently had an upper respiratory tract infection. His travel history was significant for recent deployment to Afghanistan. Blood work was unremarkable. As a part of workup, the patient underwent chest X-ray (CXR), which showed innumerable bilateral micronodules, diffuse bilateral military pattern, and right-sided pleural effusion/opacity (Figure 1). The patient then underwent a CT chest which revealed innumerable bilateral pulmonary nodules within both lung fields with a miliary type appearance; In addition, consolidative process was seen within the right middle and lower lung fields and cross table pleura with associated right-sided pleural effusion (Figures 2, 3, 4, 5). Considering his history of recent travel and characteristic CT findings suspicious for tuberculosis, he was placed in isolation. Peripheral blood smear for acid fast bacilli (AFB) was reported negative as well as the QuantiFERON tests. For the workup of pleural effusion, the patient underwent diagnostic thoracentesis. The Pleural Fluid was classified as exudative as per the Lights criteria with the cytology slides and cell block showing clusters and scattered single-lying highly atypical cells, in a background of mesothelial cells, histiocytes, and small lymphocytes. The neoplastic cells are positive for CK7, TTF-1, Napsin-A, and Ber- EP4 (focal, weak), while negative for p63, CK5/6, CD68, calretinin, WT-1, D2-40, CDX-2 and PAX-8 immunohistochemical stains, consistent with lung adenocarcinoma.

## Discussion

Lung adenocarcinoma, a subtype of NSCLC, is the most common type of lung cancer [1]. It is common in smokers as well as nonsmokers [2]. Its incidence has been increasing gradually since 1975 but with the survival been decreasing due to the introduction of low-tar filter cigarettes in the 1960s. It is more common in women as compared to men [3]. Adenocarcinoma is also the most common type of bronchogenic carcinoma in young adults (age 25 to 40 years) [4].

The most important risk factor for lung cancer is smoking and even in people who quit smoking, the risk continues to raise more as compared to those who never smoked [5]. Other risk factors include radiation therapy, environmental toxins such as asbestos, radon, metals, ionizing radiation, and polycyclic aromatic hydrocarbons [1]. The most common clinical findings are cough, hemoptysis, chest pain, dyspnea, hoarseness, pleural effusions, superior vena cava syndromes, and pan coast syndromes [6-8]. Approximately 15% to 30% of non-Asian patients and 30% to 60% of Asian patients with adenocarcinoma have a mutation of the EGFR gene [9]. The ALK gene mutations present in 2% to 7% of United States patients with NSCLC [9]. The improved response to tyrosine kinase inhibitors specific to these mutations has led the College of American Pathologists Guideline to recommend testing for EGFR and ALK mutations in all advanced-stage adenocarcinomas, mixed cancers, and those with NSCLC in whom an adenocarcinoma component cannot be excluded

CT scan is an economical, effective, non-invasive, commonly available, and quick diagnostic way for lung cancer [10]. Lung adenocarcinoma is known to have diverse CT chest findings: GGO and part-solid nodules, single or multiple solid nodules either central or peripheral, thin‐walled cystic lesion, which may be interpreted as a pulmonary cyst, bleb or bulla. Lesions are usually solitary but can also be multifocal. In a few instances, calcification or cavitation can be seen making it difficult to distinguish from infectious diseases like tuberculosis, pneumonia, or fungal infections [11] (will need more references). The adenocarcinoma’s histologic growth pattern is mostly acinar, papillary, and solid; therefore the surfaces in the lobes often have small differentiated leaves and spinous processes [10].

Our patient, presented with innumerable millet seed size solid nodules bilaterally and right lower lobe opacity associated with pleural effusion. Couple the CT findings with patient’s travel and occupation history, ruling out an infectious etiology especially military tuberculosis was the first on our list. This case adds to the pool of variable presentations in which lung adenocarcinoma can appear and easily be missed if not properly investigated.

The internists as well the pulmonologists need to be aware of atypical presentation of this subtype of NSCLC as with early-stage disease diagnosis, surgical resection is preferred. For advanced-stage lung cancer, the role of immunotherapy is promising and rapidly evolving. Checkpoint inhibitors promote recognition of cancer cells as foreign cells by the immune system and reverse the tumor-driven inhibition of the immune system that promotes tumor growth. Clinical trials using antibodies to programmed death receptor 1 and programmed death-ligand 1 have shown a significant survival benefit in advanced NSCLC [12,13].

## Conclusions

It is very important for clinicians to be aware of both typical and atypical imaging findings of lung adenocarcinoma. Our case report indicates that the index of suspicion for an atypical presentation should be very high in young adult smokers who present in a clinical setup with B symptoms (fever, weight loss, and night sweats) but have atypical imaging findings. This clinical case also emphasizes the importance of performing a biopsy and aggressively pursuing further imaging like PET scan at earlier stages of the patient encounter stage. This would help us in both diagnosing and treating such patients early. Lastly, by accurately elaborating on the radiological extent of the adenocarcinoma, appropriate and timely management of cancer can be initiated. This, if made standard at all healthcare centers, can possibly play a pivotal role in improving mortality of lung adenocarcinoma patients. 
